# The analysis of classical, polynomial regression and cubic spline mathematical models in hemp biodiesel optimization: an experimental comparison

**DOI:** 10.1007/s11356-023-31720-0

**Published:** 2024-01-08

**Authors:** Volkan Aslan

**Affiliations:** Department of Mechanical Engineering, Faculty of Engineering–Architecture, Yozgat Bozok University, Yozgat, 66200 Turkey

**Keywords:** Non-vegetable oil, Cannabis sativa, Biodiesel production, Yield, Fuel properties, Mathematical methods

## Abstract

Post-pandemic inflationist pressures, climate changes and extremes, regional conflicts, and soaring food prices caused the food crisis to increase rapidly worldwide. This global problem directs producers and researchers to use oils used as feedstock in biodiesel production effectively. In this context, it is important to assay the transesterification parameters and conduct new optimization studies to increase biodiesel yield. In this study, methyl ester was produced from hemp oil by transesterification using sodium hydroxide (NaOH). Next, classical optimization study was carried out to determine the effects of catalyst amount, alcohol:oil molar ratio, reaction temperature, and reaction time variables on biodiesel yield. Secondly, the cubic spline mathematical model (CSMM) and polynomial regression mathematical model (PRMM) were applied to the first data of this optimization. Among these optimization methods, the utmost biodiesel yield registered was 96.115% at hemp seed oil (HSO):methanol molar ratio of 5.59:1, catalyst concentration of 0.531 wt%, reaction temperature of 42.5 °C, reaction time of 62.1 min, and agitation intensity of 600 rpm at PRMM. Some vital fuel properties obtained from HSO biodiesels as a result of three optimizations satisfied the EN 14214 standard. The results illustrated that the optimal yields from CSMM and PRMM are 0.765% and 1.065% higher, respectively, according to the maximum efficiency obtained from the classical optimization. The outcomes showed that CSMM and PRMM are cost-effective, easy to handle, and promising new approaches.

## Introduction

Energy is used to meet human needs and enhance the quality of life, and it is one of the most important inputs that determine the economic and social development potential of a country (Tamilalagan et al. 2019). Nowadays, about 86% of the world’s primary energy consumption is from fossil-based sources (Mohr et al. 2015; British Petroleum 2022). However, humanity faces the hazard of depletion of fossil fuels (Srithar et al. 2017). Moreover, it is seen that global climate change and ecological balance have deteriorated due to the greenhouse gases released into the air with the energy production from these fuels (Mateus et al. 2023; Rahman and Islam 2023; Goswami and Kreith 2016). Renewable fuels significantly minimize these disadvantages and reduce foreign-source dependency by using domestic resources (Anani 2020). Biodiesel fuel is in demand owing to its convenient emission and combustion profile, carbon–neutral property, high flash point, and versatile usage. Although there are many methods to reduce the viscosity in crude oil to obtain biodiesel, transesterification is the most preferred method with its advantages (formation of products close to petrodiesel, commercialization potential, mild reaction conditions, renewability, cheap, and high conversion) (Ahmad et al. 2012; Zahan and Kano 2018). Transesterification is forming fatty ester and glycerine by reacting the triglyceride molecule with alcohol in the presence of a catalyst. Methanol and sodium hydroxide is the most chosen alcohol–catalyst binary in the transesterification reaction due to ease of handling, cheap and abundant availability, and high yield (Seffati et al. 2019; Talha and Sulaiman 2016).

Policymakers, environmentalists, and energy producers have shown great interest due to the fact that biodiesel is an environmentally friendly fuel. It is considered that an alternative energy source to fossil fuels, biodiesel is expected to play an impressive role in achieving the Sustainable Development Goals (SDGs). Biodiesel corresponds to some SDGs and supports the Paris Climate Change Agreement (Malla and Bandh 2023). Several reviews have stated that biodiesel and other biofuels contribute specifically to achieving SDG 7 (affordable, sustainable, reliable, and clean energy) and SDG 13 (struggling with climate change). However, to expand the use of biofuels, there is a need to reduce production costs and encourage their commercialization (Nazari et al. 2021). The feedstock used in biodiesel production is selected considering many factors such as local availability, cost, quality, oil yield, fuel performance, renewability, origin, and government support. A wide variety of feedstocks can be evaluated in biodiesel production, such as vegetable (edible and inedible) oils, animal fats, waste oil, and algal oils (Bukkarapu and Krishnasamy 2022; Atabani et al. 2012). Approximately 95% of the feedstock used in biodiesel production is selected from edible oils. However, using these edible oils in biodiesel production has increased the interest in non-edible vegetable oils due to food safety, the gap between supply and demand, and their uneconomical nature (Tran et al. 2021; Demirbas et al. 2016).

Hemp (*Cannabis sativa* L.) is one of the plants that accompanied humanity, dating back more than 8500 years (Deng et al. 2019). Industrial hemp is an agricultural product preferred in the production of a wide variety of products, including food and beverages, cosmetics and personal care products, nutritional supplements, fabrics and textiles, yarns and spun fibers, paper, construction and insulation materials, resin, pulp, and animal bedding, fuel (Wirtshafter 2004; Johnson 2014). This plant is one of the fastest-growing biomass and aligns with the green future goals societies and governments aim to transition to in the twenty-first century. Also, it is widely adaptable and demands very few pesticides or herbicides (Alcheikh 2015).

**Fig. 1:**
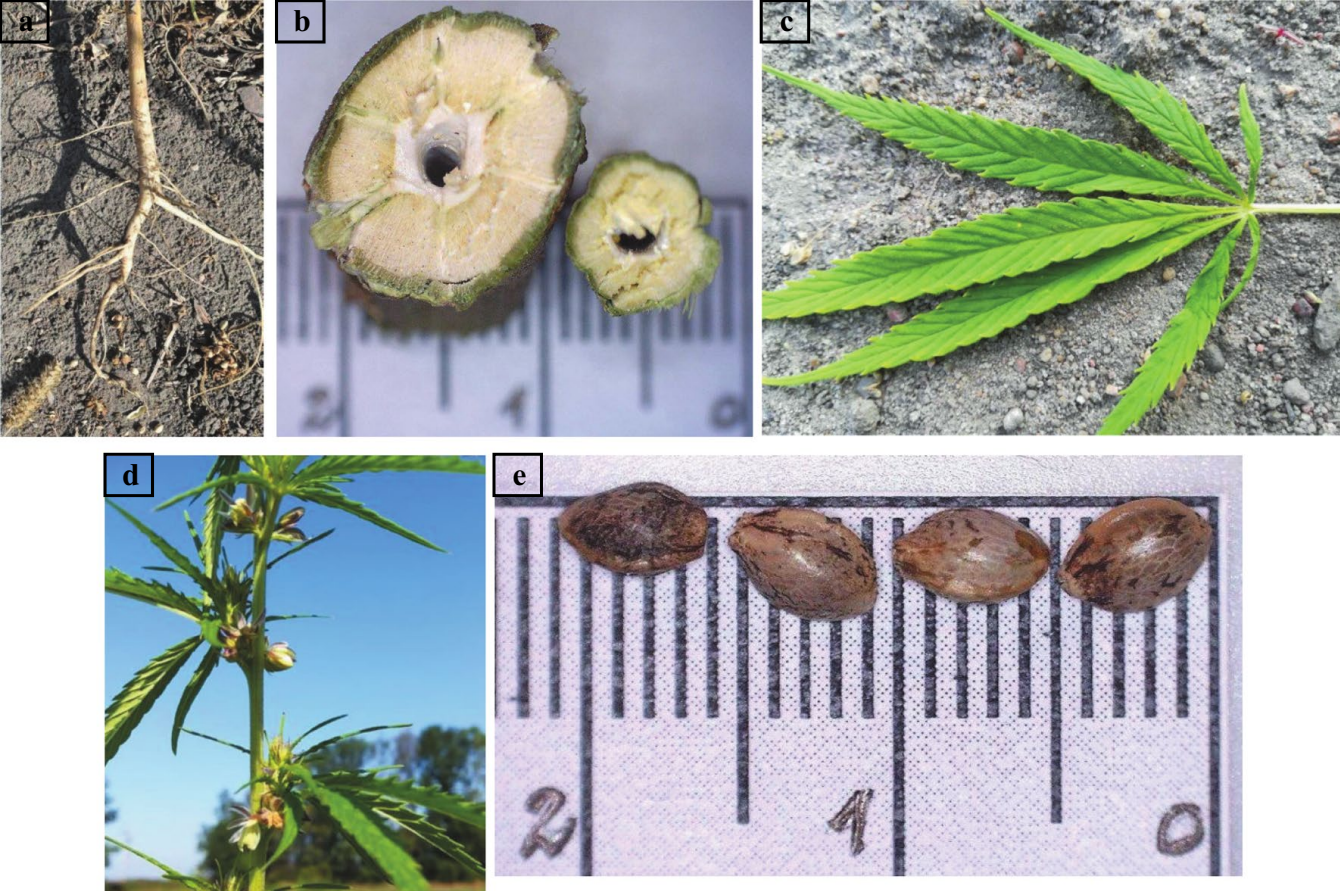
The pictures of hemp (*Cannabis sativa* L.). **a** root, **b** stem, **c** leaf, **d** flower, **e** seed (Strzelczyk et al. 2022)

Hemp has a significantly developed root system that prevents soil erosion. The stem is green, straight, and blanketed with bristle. The edge of the lance-shaped leaf is jagged, and its surface is rough. The flowers are short prongs, without petioles, and are seated. The seeds vary in diameter depending on the cannabis variety, are egg-shaped, and are between 3 and 4 mm. The weight of the seed increases as it matures and is between 8 and 26 g. The color of the seed coat is mosaic and ranges from brown to dark gray. In practice, the most desirable seed is the one with a dark and bright hue (Strzelczyk et al. 2022). The root, stem, leaf, flower, and seed picture of the hemp plant (*Cannabis sativa* L.) is illustrated in Fig. 1.

**Fig. 2:**
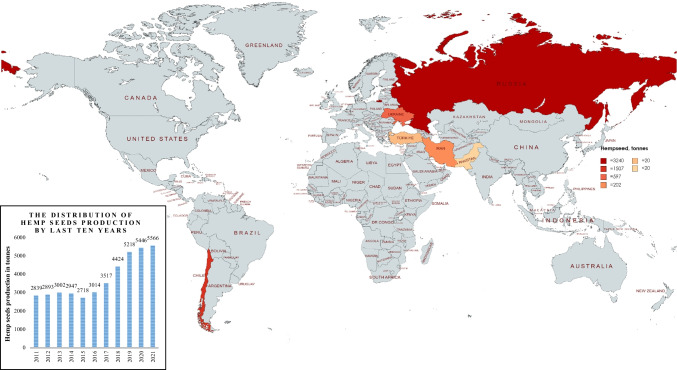
The country distributions of the annual production amount of hemp seed for 2021 year (FAO-Hempseed 2023)

According to 2021 data from the Food and Agriculture Organization (FAO), the production, area harvested, and yield of hemp seeds are around 5446 t, 10,566 ha, and 5155 hg/ha globally, respectively. For 2021, the country distributions of the annual production amount of hemp seed year were created with mapchart.net and are shown in Fig. 2. Turkey, one of the world’s cannabis producers, has recently increased its investment and support for hemp. The aim of Turkey’s recent hemp policy is to produce and expand hemp production to meet the needs of many different fields. In this context, institutes were established for research and development in cannabis production, a new cannabis variety was developed, and an action plan was made with different ministries and organizations. Yozgat Bozok University, a university in Turkey, received approval from the government as a university specializing in the field of “Industrial Hemp” in 2020 and has started research and development works.

The catalyst type and dosage, free fatty acid and moisture content, alcohol type and quantity, reaction temperature, agitation speed, and reaction time have different degrees of influence on the integrity and efficacy of the transesterification reaction. It is essential to analyze the relations of these parameters and apply the proper optimization method from the point of product efficiency and cost (Elango et al. 2019; Abbah et al. 2016). Also, selecting the optimization method implemented to these parameters is essential. Response surface methodology (Sharma et al. 2022), Taguchi (Jain et al. 2023), artificial intelligence (Said et al. 2023), and Box-Behnken (Nguyen et al. 2023) are preferred methods.

Gupta et al. (2018) evaluated the optimization of hemp (*Cannabis sativa* L.) oil transesterification variables using the Fresnel lens solar concentrator (FSC) approach and the conventional heating method approach. The reaction variables used are catalyst concentration (0.3–1.2%), alcohol:oil molar ratio (3:1–6:1), reaction time (10–80 min for conventional), and mixing speed (0–400 rpm). The yield of optimum cannabis oil methyl ester was obtained as 97.37% at 4 min reaction time, 4.5:1 methanol:oil molar ratio, 200 rpm mixing speed, and 0.90% catalyst concentration. Stamenković et al. (2015) investigated the optimization of hemp oil methanolysis production: catalyst amount (0.58–1.42 wt.% of the oil), methanol:oil molar ratio (3.5:1–8.5:1), reaction temperature (23.2–56.8 °C), and reaction time (up to 180 min). Biodiesel production process parameters were optimized using RSM and ANN combined with a genetic algorithm (GA). While the highest efficiency was 98.5% (predicted content: 99.8%) with the RSM method, it was determined as 97.5% (predicted content: 97.1%) with the ANN-GA method. Yilbasi et al. (2021) appraised using the Taguchi method (applying an L9 orthogonal design matrix) for process optimization of reaction variables: KOH catalyst concentration (0.6–1.2%), methanol/oil molar ratio (6:1–12:1), reaction time (60–120 min), and reaction temperature (30–60 °C). The optimal reaction conditions for achieving the highest FAME content (96.87%) in 120 min at the KOH loading of 0.9 (by the oil weight) was the methanol-to-oil molar ratio of 12:1 and 45 °C reaction temperature. They also stated that the methanol oil molar ratio was the most influential factor, and the reaction time was the least effective parameter in this study. Finally, Rashid et al. (2016) optimized process parameters in order to get maximum hemp seed oil (HSO) biodiesel using response surface methodology. The investigators optimized the methanol/oil molar ratio (varying from 3:1 to 9:1by steps of 1.5), NaOCH_3_ concentration (ranging from 0.25 to 1.50 wt% by steps of 0.31wt%), reaction temperature (changing from 45 to 65 °C by steps of 5 °C), reaction time (varying from 30 to 90 min of 15 min.). After all, the maximum *Cannabis sativa* oil biodiesel yield was determined by 86.01% with 7.5:1 of methanol-to-oil molar ratio, 0.80 wt% of catalyst amount, 65 min of reaction time, and 53 °C of reaction temperature.

Nowadays, food supply security, irrigation shortage, necessary energy from planting to harvesting for seeds, climate change, etc. problems are increasing daily. When these situations are taken into consideration, controlled studies should be carried out in terms of both energy and food safety policies. Feedstock cost is of great importance in biodiesel production. Also, it is essential to analyze the relations of transesterification parameters and apply the proper optimization method from the point of product efficiency and cost. In this study, the biodiesel production yield of new mathematical models was compared with the classical method. The main goal of this study is to provide the highest amount of biodiesel production using new mathematical models that are easy to use, accessible, and understandable. A comparison of the classic method and mathematical models, that are cubic spline mathematical method and polynomial regression mathematical method, for optimizing transesterification of HSO biodiesel has not been informed up to now. Thus, the present study concentrates on optimizing the transesterification of HSO for biodiesel production according to the classical optimization method by using the cubic spline mathematical method and polynomial regression mathematical method to analyze the optimal biodiesel production. At the same time, the current experimental study aims to analyze the impacts of the different process factors, including NaOH concentration, methanol:oil molar ratio, reaction temperature, and reaction time, on the conversion yield of HSO. Subsequently, the fatty acid composition and the elemental analysis of HSO biodiesel were identified. Finally, the physicochemical properties of the biodiesel fuel with the highest efficiency determined by three different optimization techniques were determined and compared using the EN14214 procedure.

## Theoretical background

In this study, in addition to the classical method, the cubic spline mathematical method (CSMM) and polynomial regression mathematical method (PRMM) optimization of the transesterification of HSO with methanol in the presence of NaOH were implemented in this work.

### CSMM

A series of continuous curves bonded to compose a single continuous curve is named a spline curve. For (*x*_*j*_, *y*_*j*_), *j* = 0,1, …, *n* – 1 and *x*_*j*_ < *x*_*j*+1_, *j* = 0,1, …, *n* considering a given set of data points; generally, *m*. degree spline *f*(*x*) for this dataset is a piecewise polynomial of degree *m* supplying the undermentioned two conditions (Maindl 2018):

1. For each interval (*x*_*j*_, *x*_*j*+1_) için, it is at *j* = 0,1, …, *n* – 1, ≤ *m* degree.
2. At each node *x*_*i*_ and in the range *j* = 1, …, *n* − 1, (*x*_0_, *x*_*n*_); the spline *f*(*x*) and its first *m* − 1 derivatives are continuous.

Cubic spline interpolation finds a curve connecting data points with three degrees or less. Splines are uniform and continuous polynomials along a given graph and are also continuous first and second derivatives where they converge. In the cubic spline approximation, the approximate cubic polynomials, the function values are such that the first and second derivatives are continuous at the nodes. Therefore, the cubic spline curve is continuous, so the gradient and curvature remain the same everywhere in the whole area. For this reason, the cubic spline provides sufficient smoothness on the approximate curve. A cubic spline polynomial *f*(*x*) provides the following three conditions (Gupta 2019):

- 1. [*x*_*j*-1_, *x*_*j*_], 1 ≤ *j* ≤ *n*, *f*(*x*) is a third-order polynomial at each subinterval.
- *f*_*j*_(*x*) = *a*_*j*_*x*^3^ + *b*_*j*_*x*^2^ + *c*_*j*_*x* + *d*_*j*_* j* = 1,2,3,….,*n*
- 2. Cubic spline values at the nodes are equal to the numerical values of the function at these points.
- 3. The polynomials $f{\prime}\left(x\right),f^{\prime\prime} \left(x\right){\mathrm{ve}}f{\prime}^{\prime\prime} \left(x\right)$ are continual along the interval (*x*_o_, *x*_*n*_).

### PRMM

The purpose of regression analysis is to estimate the parameters of the model established between the dependent variable and one or more independent variables and to determine the value of the dependent variable for the observed values of the independent variable (Freedman 2009). In some engineering calculations, the model formed by the data is unsuitable for a linear line. In such cases, using an appropriate curve for the data is necessary. At such times, the polynomial regression mathematical model is an evaluable method. The PRMM can be stated using Eqs. (1) and (2) (Aslan and Eryilmaz 2020):

1 $$f\left(x\right)={a}_{0}+{a}_{1}x+{a}_{2}{x}^{2}\cdots \cdots \cdots +{a}_{m}{x}^{m}+e$$

2 $$e=y-{a}_{0}-{a}_{1}{x}_{i}-{a}_{2}{x}_{i}^{2}\cdots \cdots \cdots -{a}_{m}{x}_{i}^{m}$$

where *y*, *x*, *m*, *e*, and *a*_0_, *a*_1_,..,*a*_*m*_ present dependent variable, independent variable, polynomial degree, error, and constant coefficients, respectively.

### Prediction of fuel properties

Biodiesel fuels with the highest biodiesel efficiency were obtained by determining the optimum values with each optimization method. The fatty acid compositions of these fuels were established via gas chromatography–mass spectrometry (GC–MS). Then, fuel properties of these biodiesels, such as density, kinematic viscosity, heating value, cetane number, flash point saponification number, iodine number, cloud point, cold filter plugging point, pour point, degree of unsaturation, and oxidation stability were estimated using the fatty acid composition values by following equations (Table 1).

**Table 1:** The equations used to predict fuel properties

Property	Formula	Definitions	Ref
Density	${\rho }_{i}=0.8463+\frac{4.9}{{{\mathrm{M}}}_{i}}+0.0118\bullet {\mathrm{N}}$	*ρ*_*i*_: density of ith fatty acid methyl ester (FAME) at 20 °C *M*_*i*_: molecular weight of ith FAME N: number of double bonds of given FAME	Ramírez-Verduzco et al. (2012)
Kinematic viscosity	${\mathrm{In}}\left({\eta }_{i}\right)=-12.503+2.496\bullet {\mathrm{In}}\left({{\mathrm{M}}}_{i}\right)-0.178\bullet {\mathrm{N}}$	*η*_*i*_: kinematic viscosity of ith FAME at 40 °C	Ramírez-Verduzco et al. (2012)
Heating value	${\updelta }_{i}=46.19-\frac{1794}{{{\mathrm{M}}}_{i}}-0.21\bullet {\mathrm{N}}$	*δ*_*i*_: heating value of ith FAME	Ramírez-Verduzco et al. (2012)
Cetane number	${\varphi }_{i}=-7.8+302\bullet {{\mathrm{M}}}_{i}-20\bullet {\mathrm{N}}$	*ϕ*_*i*_: cetane number of ith FAME	Ramírez-Verduzco et al. (2012)
Flash point	${\mathrm{FP}}=32.641\bullet \eta +305.02$	FP: flash point	Demirbas (2007)
Saponification number	${\mathrm{SN}}=\sum\limits_{i=1}^{ n }\left(\frac{ {560}\cdot {A}_{i}}{{M}_{i}}\right)$	SN:Saponification number *A*_*i*_: the percentage of each component in the fatty acid composition	Aslan (2019)
Iodine value	${\mathrm{IV}}=\sum\limits_{i=1}^{ n }\left(\frac{ 254\bullet N\cdot {A}_{i}}{{M}_{i}}\right)$	IV: iodine value	Aslan (2019)
Cloud point	$\mathrm{CP = 0.526(C16:0)}-\mathrm{4.992}$	CP: cloud point C16:0: percentage of palmitic acid	Sarin et al. (2009)
Cold filter plugging point	$\mathrm{CFPP = 3.1417(LCSF)}-16\mathrm{.477}$ $\mathrm{LCSF = 0.1(16:0\%)+0.5(C18:0\%)+}\mathrm{1(20:0\%)+1.5(22:0\%)+2(24:0\%)}$	CFPP: cold filter plugging point LCSF: long chain saturated factor	Ramos et al. (2009)
Pour point	$\mathrm{PP = }-\mathrm{30.324+0.667(16:0\%)+}\mathrm{0.4065(18:0\%)+0.11791(18:1\%)+}$ $\mathrm{+0.23225(}\mathrm{18:2\%)+0.17162(18:3\%)}-\mathrm{0.48149(22:1\%)}$	PP: pour point	Agarwal et al. (2010)
Degree of unsaturation	${\mathrm{DU}}=\sum {\mathrm{MUFA}}+2\bullet \sum {\mathrm{PUFA}}$	DU: degree of unsaturation MUFA: monounsaturated fatty acid PUFA: polyunsaturated fatty acid	Aslan (2019)
Oxidation stability	$\mathrm{OS =[(16:1\%+18:1\%+20:1\%+22:1\%}$)$+\mathrm{10.3x}\left(\mathrm{18:2\%}\right)\mathrm{+21.6x(18:3\%)]/100}$	OS: oxidation stability	Cao et al. (2015)

## Materials and methods

### Materials

#### Hemp (Cannabis sativa L.) seeds

The seeds of the “Narlıdere” type of *Cannabis sativa* (hemp seed), grown by the farmers of Yahyalı, a district of Turkey, were used. The HSO was obtained through a screw oil press machine using 8-mm nozzles. Moisture and oil contents of the seeds were determined 5.12 ± 0.11 g/100 g and 25.19 ± 0.22 g/100 g, respectively. HSO was left to rest for a few days and was filtered with Whatman filter paper for the suspended oil cake particles to settle to the bottom. Before starting the optimization studies, the titration method designated the free fatty acid content of HSO. The optimization studies applied a one-stage homogeneous catalyst transesterification process since the average FFA value (0.384%) of hemp oil is less than 1%. The average molecular weight of HSO was calculated from the distribution of fatty acid compositions with the Shimadzu single quadrupole GC–MS-QP2010 gas chromatograph–mass spectrometer.

#### Chemicals and devices

Methanol (99.8%) and NaOH (pellets pure) were procured from Merck (USA). KOH solution (0.1 N) and phenolphthalein indicator (1%) were procured from Norateks Chemical Company (Turkey), and diethyl ether was bought from Tekkim Chemical Company (Turkey). The important device of the study IKA C-MAG HS 7 (Germany) package magnetic stirrer, runs at speeds ranging from 100–1500 rpm, a heating temperature range ranging from 20–500°C, and a temperature step of 0.1°C was used. The weight measurements of the samples were made with a Weightlab LB.WL-603 (Turkey) precision balance has a readability of 0.001 g and a weighing range of up to 600 g.

### Transesterification of HSO

#### Apparatus and experimental method

**Fig. 3:**
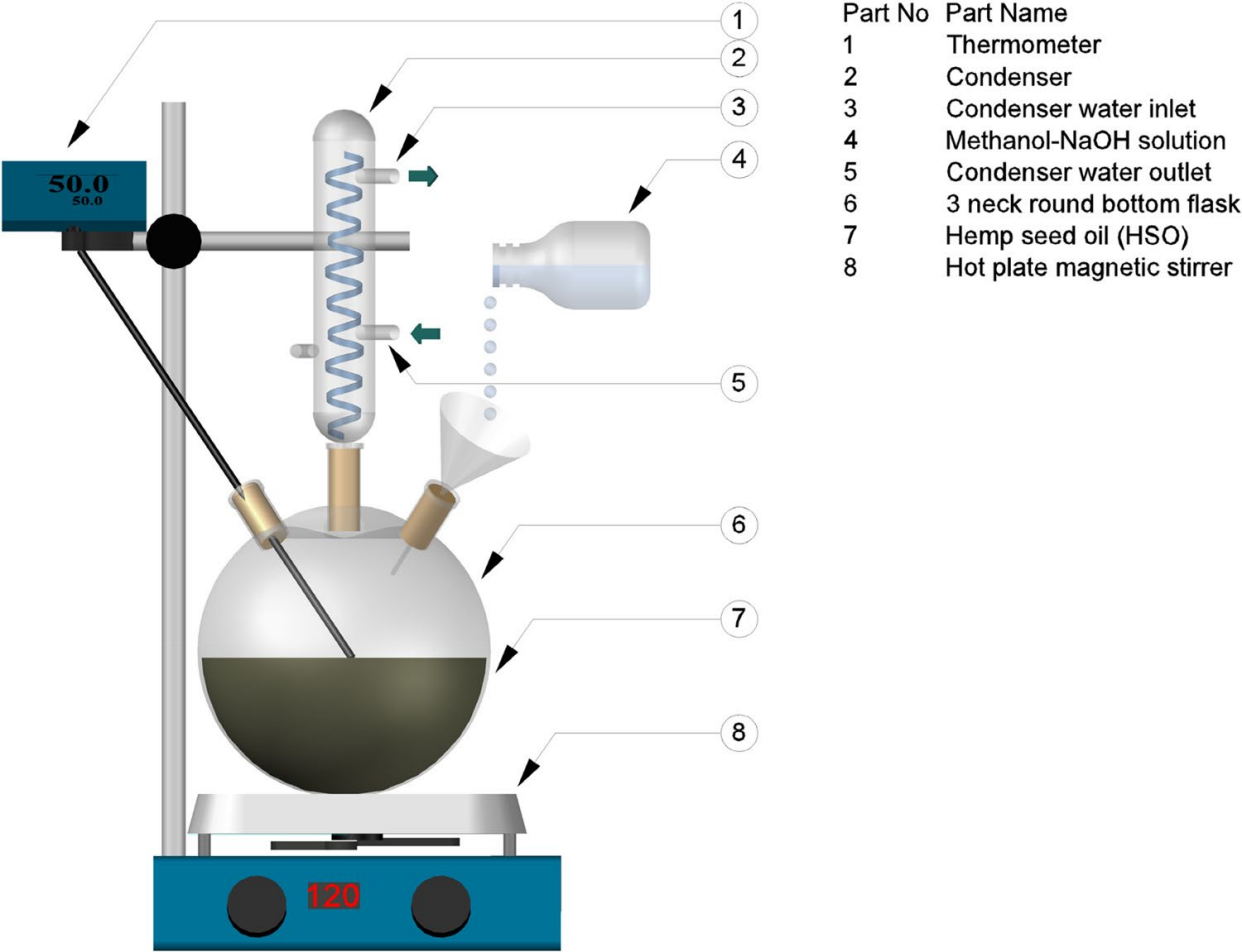
Experimental apparatus for transesterification of HSO

Each trial was carried out with 100 g ± 0.01 of HSO in optimization studies. First, the weighed and noted HSO was poured into a three-neck flat bottom, as shown in Fig. 3. Afterwards, the desired reaction temperature was adjusted, the thermometer was mounted on the magnetic stirrer with heater, and the heating and mixing processes were started. Methanol and NaOH mixed in specific proportions were mixed in a glass container until NaOH dissolved.

**Fig. 4:**
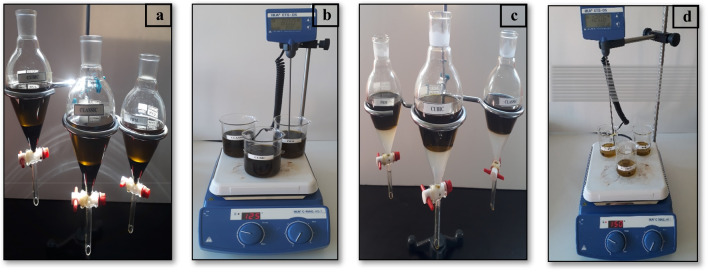
Some images related to HSO biodiesel production: **a** ester and glycerol separation, **b** alcohol evaporation, **c** purification, **d** drying

When the HSO reached the set temperature, the methanol–NaOH solution was spilt from one neck, and the time was started. The mixing intensity was fixed to 600 rpm for each trial. When the reaction time was complete, the mixture was transferred to a separatory funnel and left to rest for at least 8 h to allow the glycerol to settle to the bottom. The ester was kept in the heater for 1 h without stirring, at a temperature of 70°C (due to the boiling point of methanol) to evaporate unreacted methanol. Then, the ester was separated from glycerol and purified with distilled water. Ester and distilled water temperatures were chosen to be close to each other, between 50 and 55°C. After purification, the wastewater was taken after waiting at least 8 h. As a final process, the drying process was realized at 120°C for 2 h to remove the water in the ester. Some images related to HSO biodiesel production are presented in Fig. 4.

**Fig. 5:**
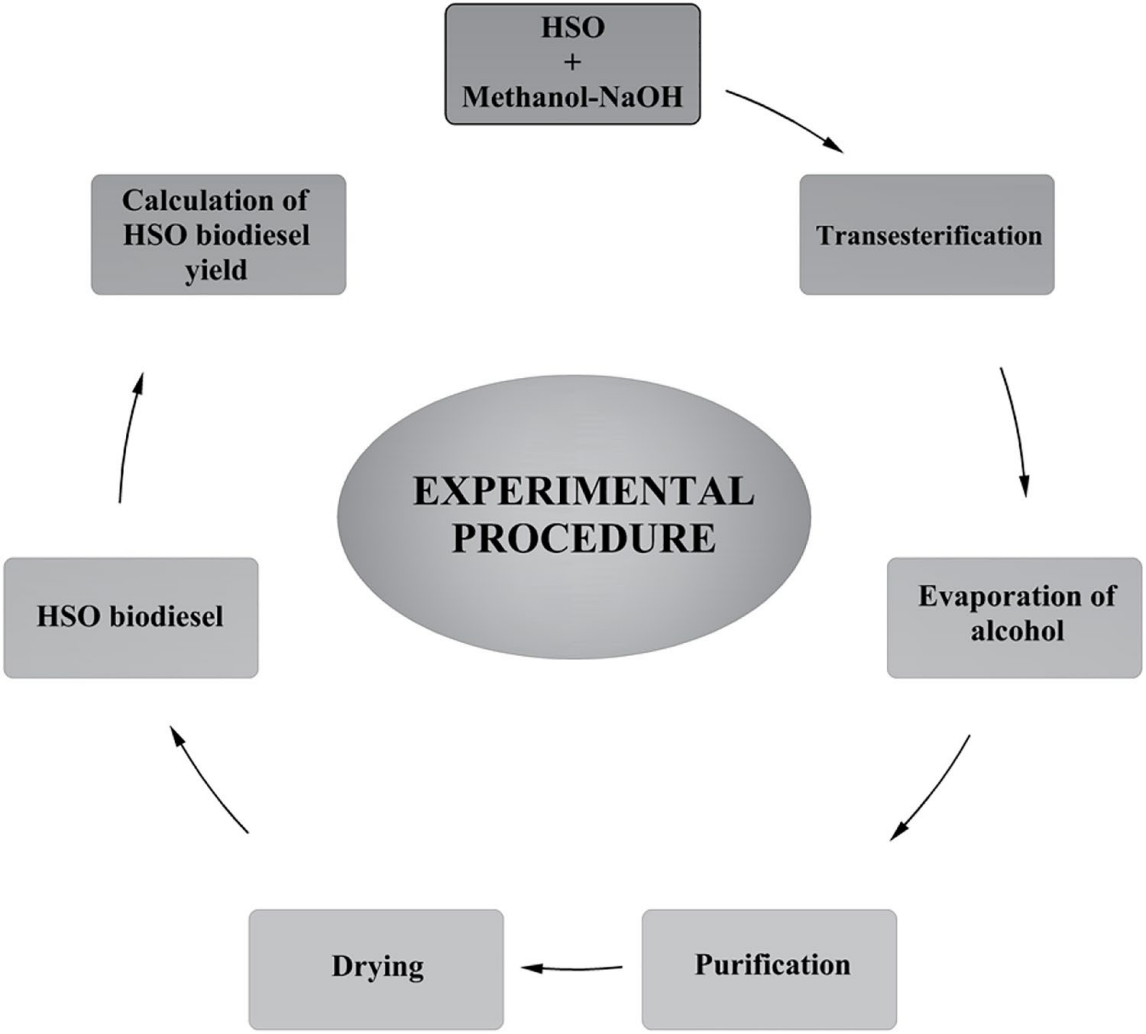
The experimental procedure for obtaining HSO biodiesel and calculating yield

The biodiesel yield at room temperature was calculated according to Eq. (3) (Dhawane et al. 2016). The experimental procedure is depicted in Fig. 5.

3 $$\mathrm{Biodiesel\, yield}\, \left(\%\right)=\frac{\mathrm{Amount\, of\, biodiesel\, produced}\,\left({\mathrm{g}}\right)}{\mathrm{Amount\, of\, oil\, used}\,\left({\mathrm{g}}\right)}\times 100$$

#### Optimization of production parameters

Optimization processes were carried out to reach the maximum ester yield with the help of CSMM and PRMM. Methanol was selected as the alcohol, and NaOH was chosen as the catalyst in optimization studies. The parameters examined using the transesterification method were catalyst concentration, methanol:HSO molar ratio, reaction temperature, and reaction time. Many researchers emphasized that examining these parameters affects transesterification (Miraculas et al. 2018; Hundie and Akuma 2022; Adenuga et al. 2020; Halwe et al. 2021). The parameter data for the optimization processes in this study are illustrated in Table 2.

**Table 2:**
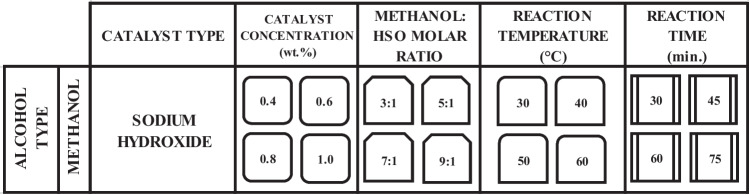
The optimization parameters and their numerical values

**Fig. 6:**
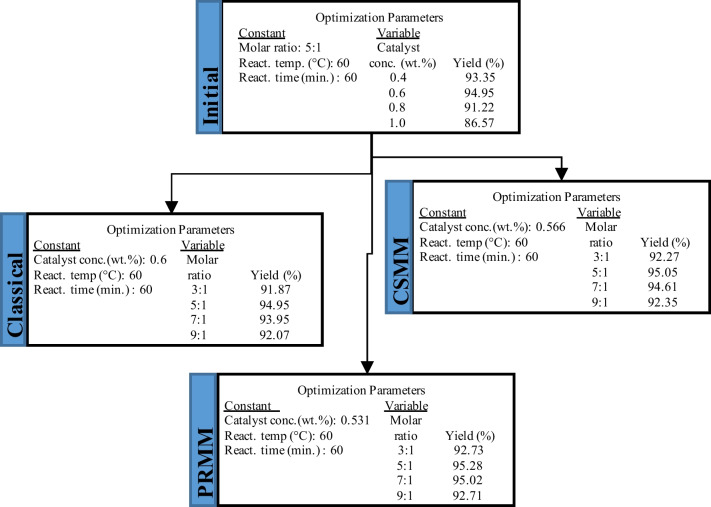
Flowchart on the use of the optimization methods

Optimization processes were started with the catalyst parameter variable and the other parameters fixed. The catalyst concentration varied from 0.4% by weight to 1.0% in 0.2% steps, while the molar ratio, reaction temperature, and reaction time were fixed at 5:1, 60 °C, and 60 min, respectively. In the classical method, the catalyst concentration with the highest efficiency was selected in the biodiesel yields obtained depending on the catalyst concentration change. This value is taken as constant in other transesterification parameter processes. The CSMM approach was applied using the biodiesel yields obtained from each parameter and the numerical variables determined for the relevant parameter. The value obtained with the CSMM application was fixed in other transesterification parameter processes. The PRMM approach pursued a similar tack to CSMM. PRMM and CSMM approaches aim to increase biodiesel production efficiency according to the classical optimization method. The flowchart relevant to using the optimization methods is illustrated in Fig. 6.

## Results and discussion

### Fatty acid composition

The fatty acid composition of HSO was analyzed by gas chromatography–mass spectrometry (GC–MS) in Yozgat Bozok University Central Laboratory to determine the molecular weight required for the methanol:HSO molar ratio. At the end of each optimization study, biodiesels with the highest yield were produced, and GC–MS determined their fatty acid distributions. The fatty acid compositions of HSO and biodiesels (obtained from classical, CSMM, and PRMM) are shown in Table 3.

**Table 3:** The fatty acid compositions of HSO and biodiesel fuels

No	Fatty acid	Molecular weight	Structure	Formula	HSO	Classical	CSMM	PRMM
1	Palmitic	256	16:0	C_16_H_32_O_2_	7.18	7.84	7.54	7.80
2	Palmitoleic	254	16:1	C_16_H_30_O_2_	0.06	0.07	0.07	0.06
3	Margaric	270	17:0	C_17_H_34_O_2_	0.04	0.03	-	0.90
4	Stearic	284	18:0	C_18_H_36_O_2_	3.21	3.72	3.89	3.69
5	Oleic	282	18:1	C_18_H_34_O_2_	18.23	19.37	19.40	19.25
6	Linoleic	280	18:2	C_18_H_32_O_2_	53.75	51.64	51.78	51.16
7	Linolenic	278	18:3	C_18_H_32_O_2_	16.55	16.09	16.10	15.74
8	Stearidonic	290	19:4	C_18_H_28_O_2_	0.22	0.22	0.19	0.21
9	Arachidic	312	20:0	C_20_H_40_O_2_	0.41	0.46	0.47	0.49
10	Behenic	340	22:0	C_22_H_44_O_2_	0.33	0.43	0.45	0.42
11	Lignoceric	368	24:0	C_24_H_48_O_2_	0.25	0.14	0.12	0.27
	ΣSFA				11.42	12.62	12.47	13.57
	ΣMUFA				18.29	19.44	19.47	19.31
	ΣPUFA				70.51	67.95	68.07	67.11

The dominant fatty acids in biodiesel produced in three optimization techniques are linoleic acid, oleic acid, and linolenic acid, similar to hemp oil, as shown in Table 2. According to the fatty acid composition, the average molecular weights of the biodiesels obtained by classical, CSMM, and PRMM were calculated as 876.10, 875.98, and 876.07 g/mol, respectively.

### Optimization of HSO biodiesel production

The experiments were conducted using classical, CSMM, and PRMM optimisation techniques. The transesterification process was optimized using four parameters: catalyst concentration (0.4–1.0 wt%), methanol:HSO molar ratio(3:1–9:1), reaction temperature (30–60°C), and reaction time (30–75 min.).

#### Effect of catalyst concentration

**Fig. 7:**
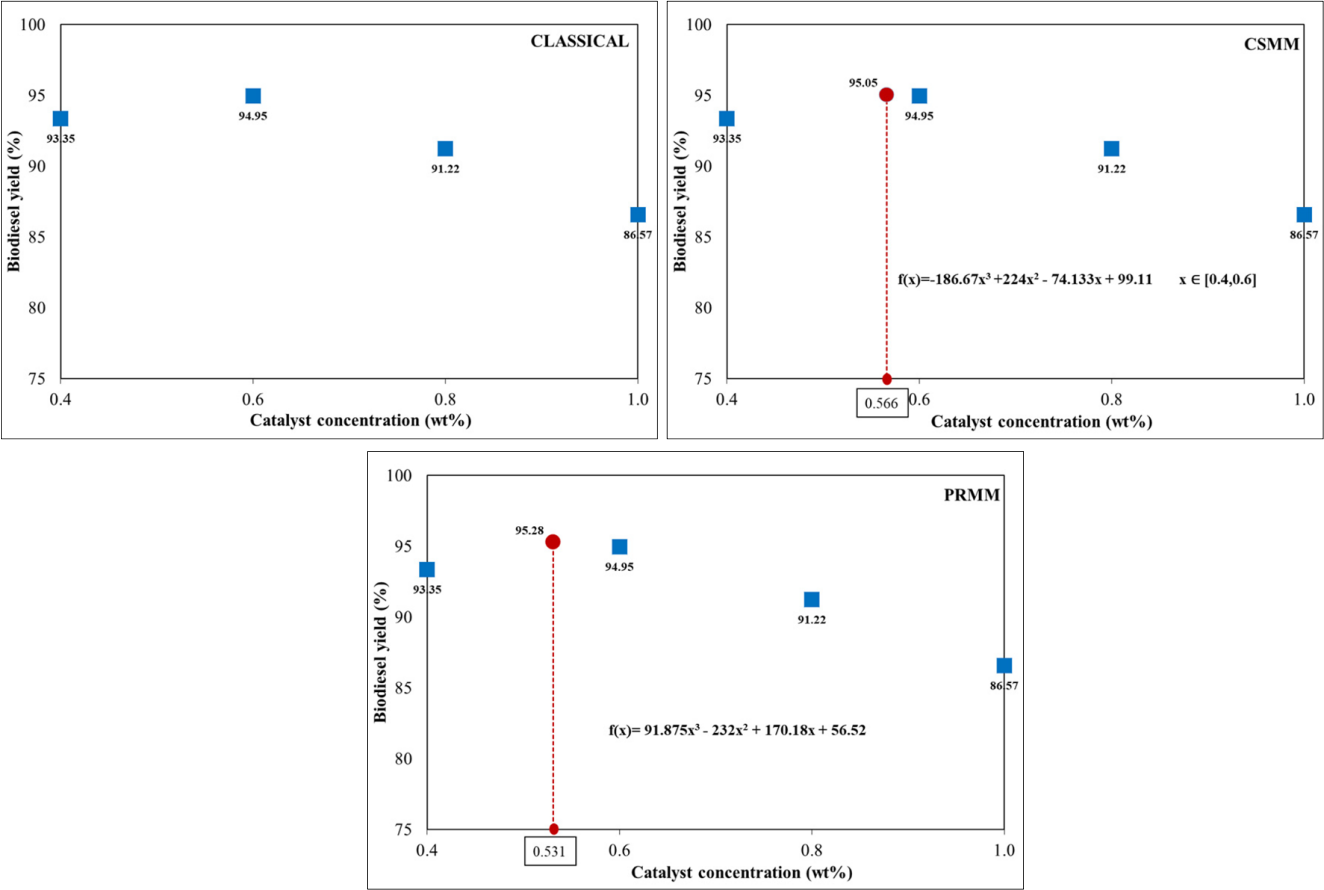
Effect of catalyst concentration on biodiesel yield for classical, CSMM, and PRMM methods

The transesterification of the HSO to biodiesel was investigated under various catalyst concentrations, 0.4–1.0%, at an interval of 0.2%, while other conditions were kept fixed: 5:1 molar ratio (methanol:HSO), 60 °C reaction temperature, 60 min reaction time, and stirring at 600 rpm. The effect of catalyst concentration for different optimization conditions is given in Fig. 7. As a result of the experiments, the highest yield was obtained with 94.95% at 0.6 catalyst concentration. These data were used in the classical method in the following reaction conditions. The cubic spline mathematical model and polynomial regression mathematical model were applied to the catalyst concentration and biodiesel yield data. The highest yields and catalyst concentrations were determined at 95.05% to 0.566 wt% and 95.28% to 0.531wt% for CSMM and PRMM, respectively.

It has been determined that the catalysts used at both high and low ratios reduce the efficiency, but there is an optimum point in the middle points. When a large catalyst dosage is utilized, the catalyst reacts with free fatty acids in the oil and form saponification. Conversely, when a low amount of catalyst is preferred, the reaction cannot be completed, and the ester is not formed (Sultana et al. 2014). From Fig. 7, it can be understood that the CSMM and PRMM applications increased biodiesel yield according to classical methods.

#### Effect of methanol:HSO molar ratio

**Fig. 8:**
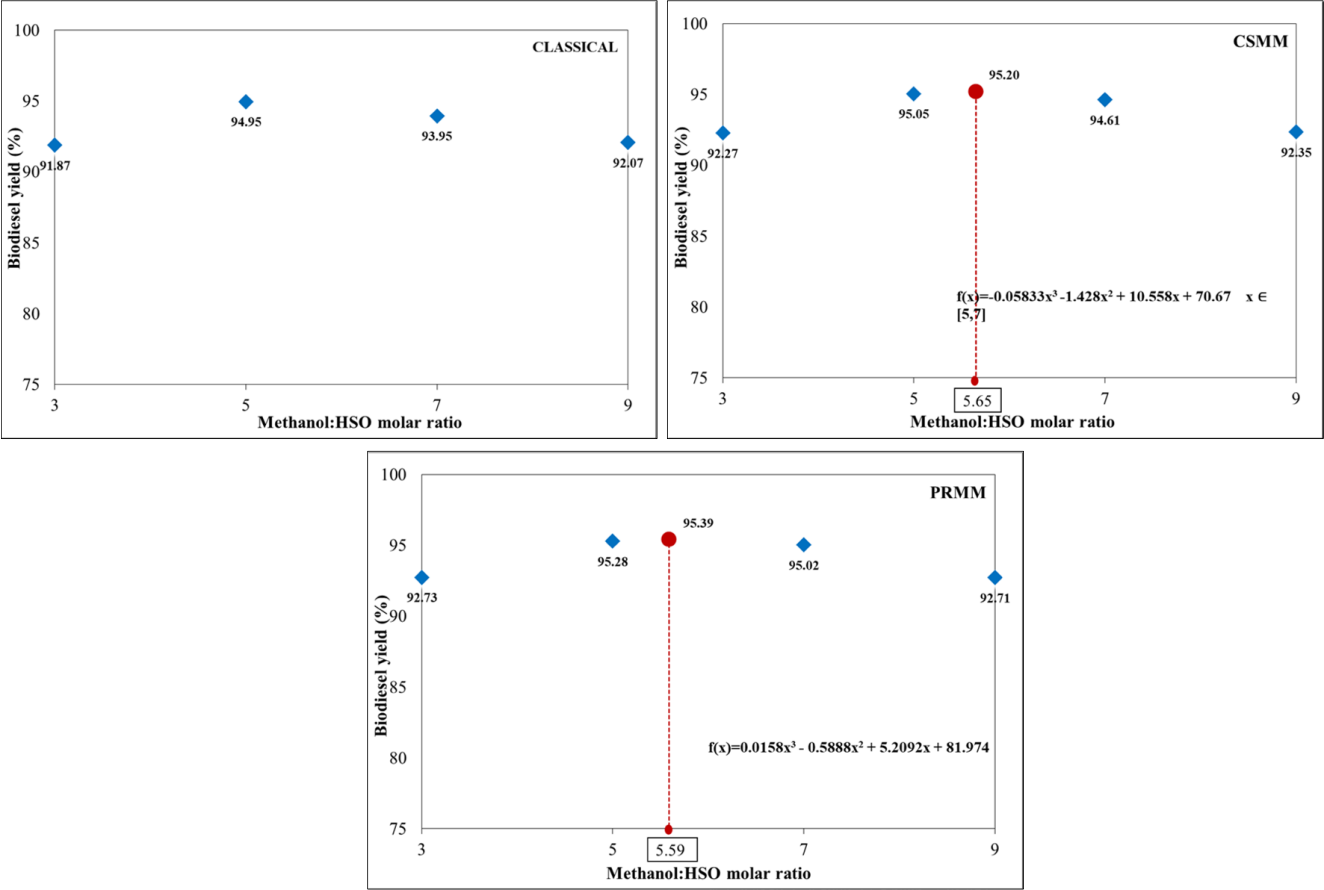
Effect of methanol:HSO molar ratio for classical, CSMM, and PRMM methods on biodiesel yield

The alcohol:oil molar ratio is one of the most critical reaction parameters affecting biodiesel yield. Although there is a 3:1 stoichiometric ratio between the oil and alcohol in the transesterification reaction, more stoichiometric ratios are needed to improve the mass transfer between the triglyceride and the alcohol molecule and to increase miscibility (Almasi et al. 2021). Methanol:HSO molar ratios of 3:1, 5:1, 7:1, and 9:1 were studied using 60 °C and 60 min stirring at 600 rpm. Concentrations of catalyst were determined in the previous parameter study. The classical method chose the catalyst concentration corresponding to the highest efficiency as 0.6 wt%. Catalyst concentrations in CSMM and PRMM applications were established as 0.566 wt% and 0.531 wt%, respectively, with the aid of the applied equations. The influence of methanol: HSO molar ratio for different optimization conditions is shown in Fig. 8.

The optimum yields and molar ratios were obtained at 94.95% to 5:1, 95.20% to 5.65:1, and 95.39% to 5.59:1 for classical, CSMM, and PRMM, respectively. In each optimization study, it was concluded that the biodiesel yield decreased at high molar ratios. This decrease in yield may be due to the dissolution of glycerine in excess alcohol. It is not easy to separate the dissolved glycerin from the product (Degfie et al. 2019).

#### Effect of reaction temperature

**Fig. 9:**
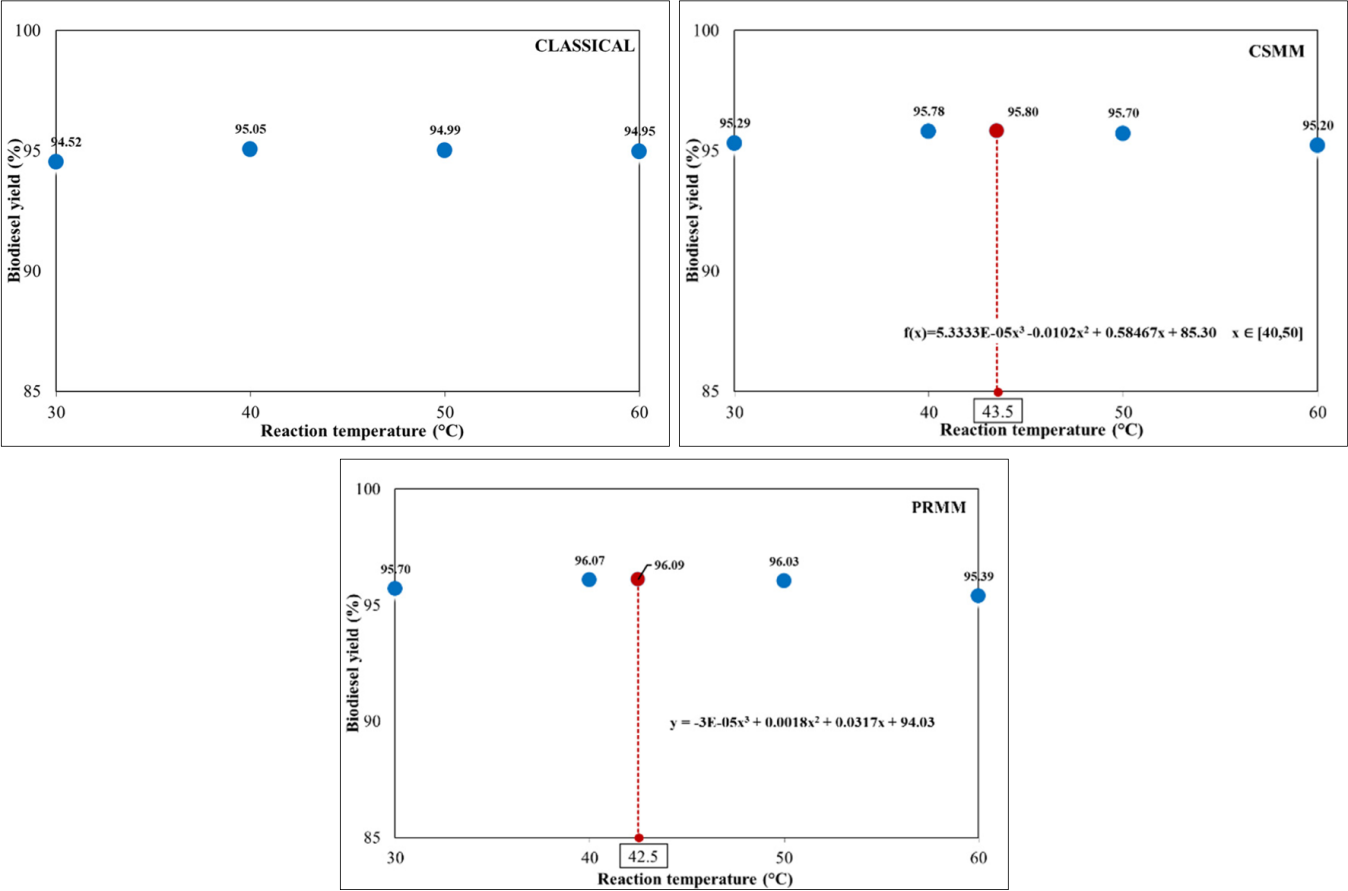
Effect of reaction temperature on biodiesel yield for classical, CSMM and PRMM methods

The transesterification process can take place at different temperatures. The reaction temperature is active in the reaction rate and biodiesel yield (Pawar et al. 2022). The reaction temperatures used for the study were 30, 40, 50, and 60 °C, with a reaction time of 60 min and agitation intensity of 600 rpm fixed. Catalyst concentrations and methanol:HSO molar ratios corresponding to the highest yield in the previous conditions were retained. For classical, CSMM, and PRMM, the catalyst concentration was 0.6, 0.566, and 0.531 wt.%, respectively; the methanol:HSO molar ratios were chosen as 5:1, 5:65, and 5:59, respectively. The effect of reaction temperature for various optimization conditions is given in Fig. 9.

A decrease in temperatures below and above 40 °C occurred. The optimum yields and reaction temperatures were attained at 95.05% to 40 °C, 95.80% to 43.5 °C, and 96.09% to 42.5 °C for classical, CSMM, and PRMM, respectively.

#### Effect of reaction time

**Fig. 10:**
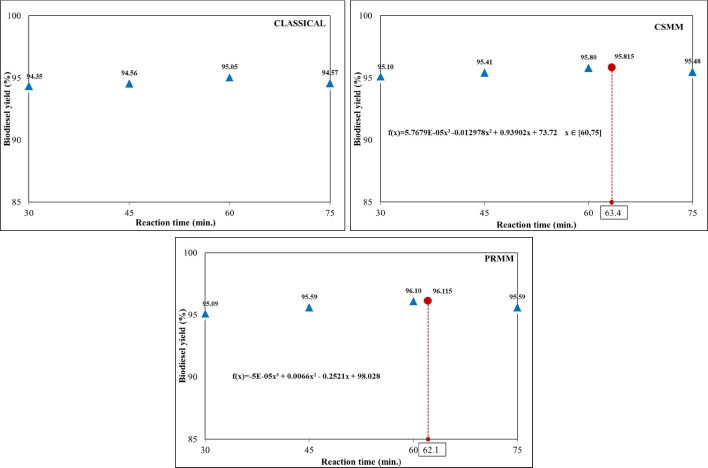
Effect of reaction time for classical, CSMM, and PRMM methods on biodiesel yield

The methyl ester yield increases as the reaction time increases to a certain point (optimum point). The reaction time beyond this point does not increase the conversion rate due to supporting the back reaction (hydrolysis of esters) (Lakshmana Naik et al. 2015). Aslan and Eryilmaz (2020) stated that 1 h might be appropriate for the optimal reaction time for the ester conversion**.** The values at the optimum points determined in the previous stages were used as the data of other parameters except for the reaction time. For classical, CSMM, and PRMM, the catalyst concentration was 0.6, 0.566, and 0.531, respectively; the molar ratios of methanol:HSO were chosen as 5:1, 5:65, and 5:59, respectively; the reaction temperature was taken 40, 43.5, 42.5 °C, respectively. The influence of reaction time for classical, CSMM, and PRMM is given in Fig. 10. The yields peaked at around 60 min and then started to decline. The highest yields and reaction times were determined at 95.05% to 60 min, 95.815% to 63.4 min, and 96.115% to 62.1 min for classical, CSMM, and PRMM, respectively.

#### Optimum transesterification parameter conditions and characterization of biodiesels

The optimal conditions for obtaining the maximum yield for each optimization as a result of three different optimization processes are given in Table 4. When the results are examined, the data obtained from the classical method are compatible with the results of a previously reported study in the literature (Ahmad et al. 2011). While the highest efficiency is obtained with the PRMM method at 1.065% compared to the classical method, this increase is 0.765% in the CSMM method.

**Table 4:** Optimum reaction conditions of classical, CSMM, and PRMM methods

Methods	Optimal condition variables				
	Catalyst concentration (wt%)	Methanol:HSO molar ratio	Reaction temperature (°C)	Reaction time (min)	Yield (%)
Classical	0.600	5.00:1	40.0	60.0	95.050
CSMM	0.566	5.65:1	43.5	63.4	95.815
PRMM	0.531	5.59:1	42.5	62.1	96.115

The HSO biodiesel produced by the transesterification reaction was characterized to determine its physicochemical properties such as density, kinematic viscosity, calorific value, cetane number, saponification number, iodine number, flash point, cloud point, cold filter plugging point, pour point, unsaturation degree, and oxidation stability and shown in Table 5. According to the fatty acid compositions given in Table 3, the fuel properties of biodiesels were estimated with the aid of the equations given in the subsection “Prediction of fuel properties.”

**Table 5:** The fuel properties of biodiesels derived from classical, CSMM, and PRMM methods

			EN 14214				
No	Property	Unit	Min	Max	Classical	CSMM	PRMM
1	Density at 20 °C	kg/m^3^	860	900	876.10	875.98	883.80
2	Kinematic viscosity at 40 °C	mm^2^/s	3.5	5.0	3.48	3.48	3.49
3	Calorific value	MJ/kg	-	-	39.405	39.402	39.402
4	Flash point	°C	101	-	145	145	146
5	Acid value	mg KOH/g	-	0.50	0.82	0.98	0.79
6	Cetane number	-	51	-	38.435	38.600	38.888
7	Iodine value	g I_2_/100 g	-	120	155.82	155.09	155.84
8	Saponification value	mg KOH/g	-	-	200.69	200.68	200.64
9	Polyunsaturated methyl ester (≥ 4 double bonds)	% (m/m)	-	1	0.22	0.19	0.21
10	Degree of unsaturation^5^	-	-	-	154.9	154.43	153.11
11	Oxidation stability value, COX	h	-	> 6	8.99	8.94	8.86
12	Cloud Point, CP	°C	Report		− 0.80	− 0.80	− 0.88
13	Pour Point, PP	°C	-	-	− 7.76	− 7.69	− 7.79
14	Cold filter plugging point, CFPP	°C	Report		− 3.82	− 3.76	− 3.84
15	Alkali content (Na + K)	mg/kg	-	5.0	0.541	1.378	1.121
16	Alkaline-earth content (Ca + Mg)	mg/kg	-	5.0	9.647	6.758	6.870
17	Phosphorus content	mg/kg	-	4.0	n.d	n.d	n.d

Table 5 presents that iodine number, acid number, and alkaline–earth content values are higher than the upper limit of the standard value. On the contrary, the cetane number values are lower than the sublimit of the standard value. The higher the number of double bonds, the higher the iodine number. The iodine number is also related to the cetane number. High unsaturation causes the decrease of cetane number in biodiesel (Yesilyurt et al. 2020).

## Conclusion

This work optimizes the condition variables using classical, CSMM, and PRMM methods. The results show that parameters other than reaction time have an important effect on biodiesel yield. The maximum biodiesel yield of 96.115% was achieved with NaOH concentration (0.531 wt%), HSO:methanol molar ratio (5.59:1), reaction temperature (42.5 °C), and reaction time (62.1 min) using PRMM method. Compared with the classical method, the biodiesel yield of the CSMM and PRMM methods are increased by 0.765% and 1.065%, respectively. Meanwhile, the different models based on third-degree polynomial equations were generated to reflect the correlation between each optimization parameter and biodiesel yield, with the coefficient of uncertainty (*R*^2^) reaching 0.9999 and 1.0000, respectively. All the specified PRMM models showed successfully estimated capability. In this period of the global food crisis, CSMM and PRMM optimization techniques have proven to be good alternatives in biodiesel production.

## Author ccontributions

Material preparation, data collection, and analysis were performed by Volkan Aslan. The first draft of the manuscript was written by Volkan Aslan, and conceptualization, methodology, validation, project administration, supervision, writing—review and editing were performed by Volkan Aslan. Volkan Aslan approved the final manuscript.

## Data Availability

The datasets used and/or analyzed during the current study are available from the corresponding author on reasonable request.
